# A physics-inspired memory-augmented deep learning framework for magnetic core loss prediction

**DOI:** 10.1371/journal.pone.0339490

**Published:** 2026-01-13

**Authors:** Haifang Cong, Siyu Chen, Yang Yang, Tianyun Luan, Chao Yang

**Affiliations:** 1 Changchun University of Science and Technology, Changchun, China; 2 Changchun Sci-Tech University, Changchun, China; 3 Beijing Aerospace Times Laser Navigation Technology Co., Ltd, Beijing, China; Guangdong University of Petrochemical Technology, CHINA

## Abstract

Accurate prediction of magnetic core loss is a key challenge for improving the efficiency and reliability of power electronic systems. Traditional empirical models such as the Steinmetz equation are only applicable to sinusoidal steady-state conditions and struggle with the complex non-sinusoidal waveforms and variable operating conditions in modern power electronics. While existing deep learning methods have shown improvements, they still face fundamental limitations in handling the nonlinear mismatch between B(t) and H(t) waveforms, coupling of multi-scale loss mechanisms, and generalization under extreme operating conditions. This paper proposes an Enhanced Memory Augmented Mamba (EMA-Mamba) model that achieves breakthrough progress in magnetic core loss prediction. It utilizes a state-space memory augmentation mechanism that stores and retrieves typical magnetization patterns through a trainable external memory matrix, endowing the model with a capability similar to the “magnetic memory” of magnetic materials, effectively solving the gradient vanishing problem in long sequence modeling. Combined with an attention-guided intelligent feature selection mechanism, it adaptively identifies critical turning points in hysteresis curves through a Top-K strategy, fundamentally solving the temporal mismatch problem between B(t) and H(t) waveforms. Finally, through a physics-constrained multi-objective optimization framework, it achieves decoupled modeling of hysteresis loss, eddy current loss, and residual loss through loss function combination, overcoming the optimization difficulties caused by data spanning six orders of magnitude. Experiments on the MagNet dataset containing 10 materials and over 150,000 data points show that EMA-Mamba achieves an average prediction error of 4.50% and a coefficient of determination of 99.9947%, reducing error by 34.2% compared to state-of-the-art baseline methods, with a 36.2% reduction in 95th percentile error under extreme conditions. The model demonstrates excellent temperature robustness and cross-material generalization capability, providing a reliable theoretical tool for intelligent design and optimization of magnetic components.

## Introduction

In modern power electronics and magnetic component design, the accuracy of core loss models has a decisive impact on system performance. With the rapid development of renewable energy, electric vehicles, and high-efficiency power conversion systems, the demand for high-performance magnetic materials and accurate loss prediction models has become unprecedentedly urgent [1]. Accurate prediction of core loss can not only improve system efficiency and reduce energy loss but also extend equipment life and enhance reliability, which has significant economic and social value in practical applications.

However, the nonlinear characteristics, frequency dependence, temperature sensitivity, and complex multivariable influencing factors of magnetic core materials make loss modeling an extremely challenging task [2]. Traditional loss models, such as the Steinmetz equation (SE) [3], have been widely used due to their simplicity and computational efficiency. However, SE is only applicable to sinusoidal steady-state conditions, and its accuracy significantly decreases for non-sinusoidal, pulsed, and high-frequency waveforms common in modern power electronics [4]. To address this, researchers have made multiple improvements to SE, proposing modified SE (MSE) [5], generalized SE (GSE) [6], improved generalized SE (iGSE) [7,8], and other models to expand their applicability.

Although these improved models have enhanced prediction accuracy to some extent, traditional models typically only consider single variables such as frequency or flux density amplitude in cross-coupling effects, lacking description of interactions between multiple factors, making it difficult to accurately reflect loss characteristics under actual operating conditions [9]. These models require predetermined material characteristic parameters, such as Steinmetz coefficients, which can usually only be obtained through experimental measurements, limiting the model’s universality and adaptability [10]. Under non-sinusoidal or complex waveform conditions, the accuracy of traditional models significantly decreases, failing to meet the needs of modern power electronics applications [11].

With the development of power electronics technology, the operating conditions of magnetic components have become increasingly complex, and traditional models struggle to adjust and adapt in time, limiting their application in emerging fields [12]. To address these issues, researchers have begun exploring data-driven approaches. Artificial neural networks (ANNs), with their powerful nonlinear mapping capability and adaptive learning characteristics, have become powerful tools for core loss modeling [13]. In early work, Kucuk [14] used neural networks and genetic algorithms to predict magnetic core hysteresis loops; Amoiralis et al. [15] combined artificial intelligence technology with hybrid finite element-boundary element methods to achieve global optimization design of transformers.

In recent years, with the development of deep learning, more researchers have turned their attention to more complex neural network models. Haoran Li et al. [16] used two-stage network regression to predict core loss, achieving accurate modeling of core loss in specific applications, but this method relies heavily on material-specific training data and lacks generalization across different core materials.Dogariu et al. [17] utilized transfer learning techniques to significantly reduce the amount of training data required for remodeling, improving model practicality, though the transfer performance degrades significantly when the target domain differs substantially from the source domain. Serrano et al. [18] proposed an encoder-decoder architecture based on long short-term memory networks (LSTM), learning magnetic field intensity sequences H(t) from flux density sequences B(t), improving the ability to capture dynamic characteristics, however, LSTM struggles with capturing long-range temporal dependencies in extended sequences due to gradient vanishing issues.Although neural network methods have improved model accuracy and applicability to some extent, they have also brought new challenges. Many models only utilize single types of data (such as scalars or time series) and cannot comprehensively capture the complex multi-scale characteristics of core loss [19]. When facing multimodal learning to fuse different modal data, reasonable weight allocation for each modality is needed to avoid information imbalance [20]. The need to simultaneously process features at different scales such as time, frequency, and space increases the complexity of model design and training [21]. To facilitate practical applications, H(t) is restricted from being directly used as input in both traditional equation-based and neural network-based core loss models [22], leading to incomplete feature learning by the model.

In response to these issues, physics-inspired modeling methods have gradually gained attention. These methods attempt to incorporate the physical properties of magnetic materials into models, reducing dependence on large amounts of training data and improving model generalization capability. Bar’yakhtar and Ivanov [23] reviewed the history and applications of the Landau-Lifshitz-Gilbert (LLG) equation, demonstrating the possibility of applying micromagnetic theory to macroscopic loss calculations. Tanaka et al. [24] incorporated the LLG equation into magnetic circuit models, achieving iron loss calculations. Saeed et al. [25] simulated variable magnetic elements including hysteresis and eddy current losses, providing new ideas for physics-inspired loss modeling. Additionally, the successful application of multimodal and hybrid methods in other fields has also provided inspiration for core loss modeling. Wang et al. [26] proposed a three-stage multimodal data fusion method for bearing fault diagnosis, achieving accurate identification of complex fault patterns. Yang et al. [27] used contrastive learning for multimodal data fusion in violence detection, significantly improving model robustness and accuracy.

**Fig 1:**
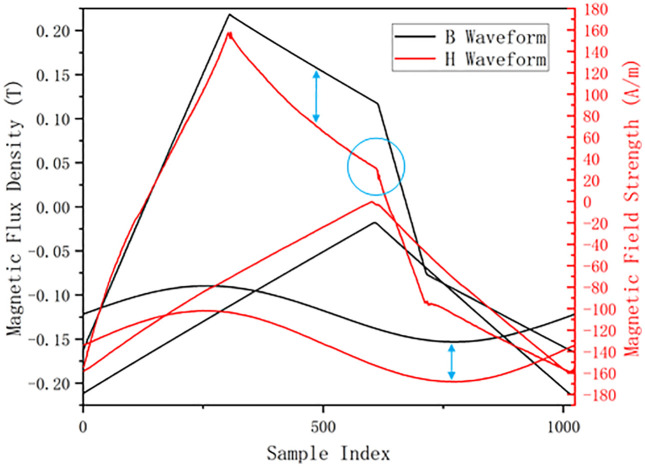
Node trend sequence mismatch problem between B(t) and H(t).

In summary, although extensive research has been devoted to core loss modeling, existing methods still struggle to comprehensively address challenges such as complex nonlinearity and sequence mismatch between B(t) and H(t) (as shown in Fig 1). To address these issues, we propose a novel model called Enhanced Memory Augmented Mamba (EMA-Mamba). Our work aims to:

1. **State-space memory-augmented long-range dependency modeling theory**

We combine Mamba’s continuous state-space modeling capability with an external memory augmentation mechanism, constructing a unified architecture that can simultaneously handle local temporal dependencies and global feature memory by enhancing Mamba’s memory capability. Through designing a trainable memory matrix $M\in R^{\wedge }(m\times d)$ and bidirectional softmax similarity computation mechanism, the model can adaptively store and retrieve typical magnetization patterns, thereby introducing a mechanism similar to the “magnetic memory” of magnetic materials in temporal modeling, reducing the computational complexity of long sequence processing and providing a physics-inspired memory mechanism that enables it to “learn to remember” typical magnetization behavior patterns under different operating conditions.

2. **Intelligent feature decoupling theory for multimodal temporal alignment**

Addressing the essential mismatch problem between B(t) and H(t) waveforms, we propose an attention-guided Top-K feature selection and multi-scale fusion strategy that understands overall trends while distinguishing waveform details and learning their implicit correspondence in high-dimensional feature space. Through attention weights adaptively identifying key time points in hysteresis curves, the model can focus on key magnetization states that determine loss characteristics without losing global information, solving the limitations of traditional point-to-point mapping methods and providing new input information and differentiation processes for handling alignment problems of heterogeneous temporal data.

3. **Physics-constrained multi-objective collaborative optimization theory**

Traditional single loss function optimization methods cannot effectively handle the coupling effects of multiple physical mechanisms in core loss. The complexity of core loss stems from the nonlinear coupling of hysteresis loss, eddy current loss, and residual loss at different spatiotemporal scales. This multi-mechanism coexistence requires optimization objectives to ensure overall prediction accuracy while applying specialized constraints to each physical mechanism during training. Therefore, we innovatively propose a hierarchical optimization decoupling framework based on magnetic physics constraints, transforming this physical understanding into mathematical constraints to construct a multi-layer progressive loss function system, avoiding the problem of mutual interference between different loss mechanisms in traditional methods. More importantly, it establishes a direct mapping channel from micromagnetic theory to macroscopic loss prediction, providing a new theoretical paradigm for physics-driven deep learning modeling.

This research proposes a new core loss modeling paradigm that achieves effective bridging from micromagnetic theory to macroscopic engineering applications. By organically combining state-space modeling, memory augmentation, and physical constraints, EMA-Mamba not only solves the prediction accuracy problem of traditional methods under complex operating conditions but also opens new research directions for intelligent modeling of magnetic materials.

### Datasets

This research adopts the MagNet open-source database jointly developed by Princeton University and Dartmouth College as the fundamental research platform. The database contains over 150,000 precisely measured excitation waveform data points for ferrite materials, covering various waveform types in the frequency range of 50–500 kHz and magnetic flux density range of 10–300 mT. The MagNet database was designed with the concept of breaking through the limitations of traditional Steinmetz equations, combining large-scale experimental data with artificial intelligence methods to provide a solid data foundation and validation environment for our proposed novel core loss modeling method. Through in-depth analysis of the B-H cycle characteristics and hysteresis loss performance of TDK, Ferroxcube, and Fair-Rite series materials in the database, this research reveals key magnetic parameter interdependencies neglected by traditional modeling methods, successfully constructing a comprehensive model capable of accurately predicting core loss under different operating conditions. This model not only overcomes the limitations of existing methods in handling waveform diversity and material nonlinearity but also provides more efficient and accurate analytical tools for power electronic systems and magnetic component design, with significant importance for improving power system efficiency and reliability.

**Fig 2:**
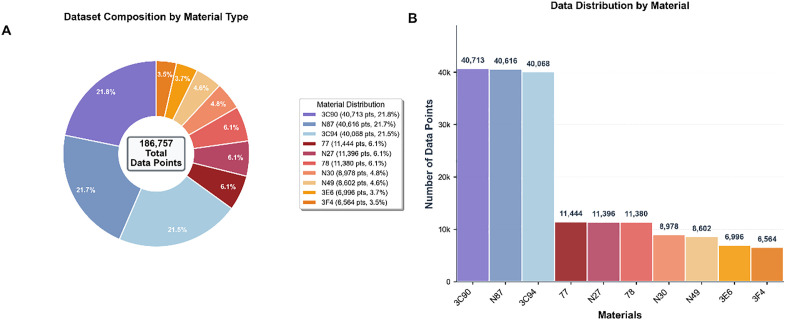
Statistical analysis of MagNet dataset.

The MagNet dataset exhibits a significant head effect in the statistical characteristics shown in Fig 2. Three materials—3C90, N87, and 3C94—account for nearly 65% of the total sample size, with each material contributing over 40,000 data points, forming the main body of the dataset. The sample sizes of the remaining seven materials show a stepwise decline. This long-tail distribution imposes higher requirements on the model’s generalization capability. Therefore, the model needs to have corresponding features to meet the requirement of enhanced characteristic memory for minority materials.

### Pre-experiment

**Fig 3:**
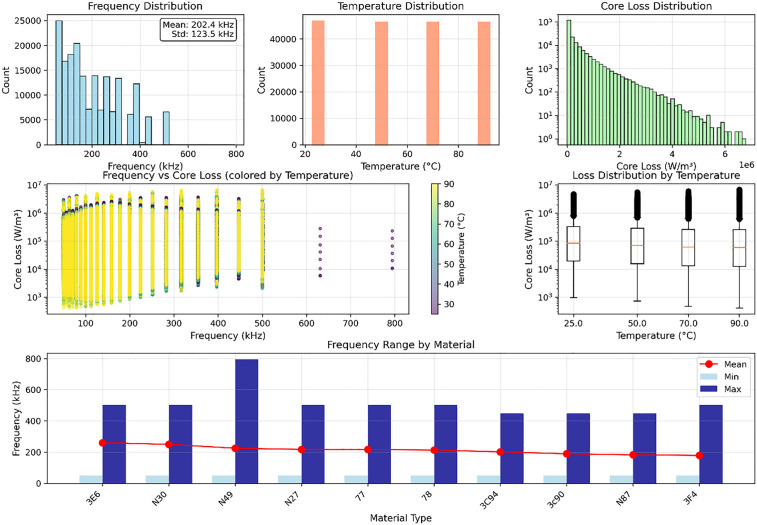
MagNet data distribution illustration.

Before formal experiments, we conducted pre-experimental analysis on MagNet to initially observe and analyze the dataset composition and characteristics, as shown in Fig 3. Statistically, the dataset presents complex multidimensional features and high heterogeneity. The frequency dimension shows obvious non-uniform distribution characteristics, while the temperature distribution shows discrete characteristics with four standard test temperature points: 25°C, 50°C, 70°C, and 90°C, with basically balanced sample sizes at each temperature point, facilitating comparative analysis of material characteristics under different temperature conditions. The core loss distribution exhibits typical log-normal distribution characteristics, with loss values spanning a wide range from 10² to 10⁶ W/m³. Low-loss region samples dominate in quantity while high-loss samples are relatively scarce. The introduction of temperature gradients causes the frequency-core loss 2D distribution scatter plot to show clear stratified structure, with high-temperature data points mainly distributed in medium-high loss value regions, while low-temperature samples concentrate more in low-loss intervals. The median loss value gradually increases with rising temperature, and data dispersion under high-temperature conditions also increases significantly. Inter-material differences also bring difficulties to core loss modeling.

**Fig 4:**
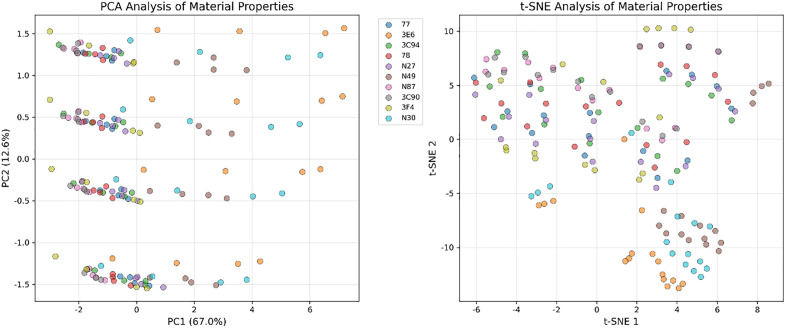
PCA and t-SNE dimensionality reduction distribution of MagNet dataset samples.

As shown in the PCA and t-SNE dimensionality reduction results in Fig 4, there exists an obvious morphological similarity hierarchical structure in the magnetic material characteristic space, reflecting deep physical correlations and common magnetization mechanisms between materials. From the overall distribution pattern, material characteristics are not randomly distributed in high-dimensional space but show obvious clustering and gradual transition features. This ordered spatial organization structure implies that the macroscopic loss behavior of magnetic materials is constrained by common rules. Taking the PCA analysis structure as an example, although there are many material types, they show significant morphological convergence in the main variation directions. Most materials’ data points are distributed along similar elliptical trajectories, differing only in the ellipse’s aspect ratio and tilt angle. This morphological convergence indicates that different materials follow similar response patterns when facing frequency and temperature changes. Although their B(t) and H(t) waveforms differ in amplitude and phase, they have intrinsic consistency in the basic laws of dynamic evolution. The t-SNE analysis results show continuous morphological transition zones and clear similarity boundaries between materials. Materials with similar magnetic properties form tight clustering clusters in t-SNE space, while materials with larger performance differences are connected through gradual transition regions. This continuous distribution reflects certain shared constraint characteristics of magnetic materials.

**Fig 5:**
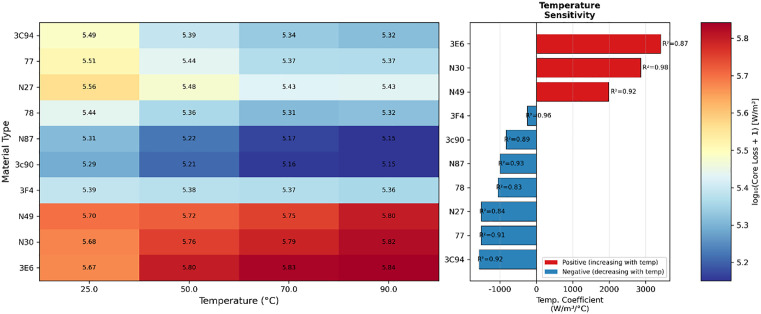
Comparison of material and temperature characteristics.

Furthermore, we plot the comparison of each material’s temperature characteristics as shown in Fig 5. There are obvious grouping phenomena in temperature response patterns between materials. Materials with similar magnetic performance parameters often exhibit similar temperature sensitivity distribution patterns. For example, materials 3E6, N30, and N49 show similar reactions to temperature characteristics, presenting positive temperature coefficient characteristics with loss decreasing as temperature increases. In the t-SNE and PCA plots, these three materials also show highly consistent distribution characteristics, presenting similar color change patterns in the heat map, indicating that these materials may have the same dominant loss mechanism and similar microstructural features, following the same regulatory constraints in macroscopic performance.

Therefore, we summarize the MagNet dataset characteristics as follows:

1. **Strong multi-variable coupling and morphological similarity**

Multi-variable strong coupling essentially stems from the phase lag effect of hysteresis phenomena. The asynchrony of B(t) and H(t) sequences in the time domain causes traditional point-to-point mapping to fail. Data shows that the nonlinear coupling in the frequency-loss-temperature three-dimensional space presents obvious stratified clustering characteristics, where single variables cannot independently predict loss behavior. High-temperature data points are mainly distributed in medium-high loss value regions, while low-temperature samples concentrate more in low-loss intervals. This temperature-induced stratified structure interweaves with material morphological similarity, forming a complex multidimensional coupling network, leading to the inability of fewer variables to independently and accurately predict loss behavior.

2. **Wide nonlinear dynamic range**

The difficulty in core loss distribution lies in the multi-scale coexistence of loss mechanisms: hysteresis loss dominates in the low-frequency band, eddy current loss surges in the high-frequency band, and complex coupling transitions exist in the mid-frequency band. The six-order magnitude loss span makes the gradient contributions of traditional loss functions extremely unbalanced across different intervals, causing models to bias toward high-loss samples while ignoring low-loss accuracy. Meanwhile, materials show obvious long-tail distribution, with extremely rich samples in the low-frequency-low-loss-major material combination space, while relatively scarce samples in the high-frequency-high-loss-rare material combination space. This multi-scale distribution imbalance causes models to easily bias toward data-rich regions during training while lacking sufficient learning capability for rare but important boundary conditions and abnormal operating conditions. Traditional single loss functions cannot effectively balance this complex distribution imbalance.

3. **Strong material and operating condition dependence**

Material and operating condition dependence reflects the interweaving of multiple complexities: different material types have distinctly different intrinsic magnetic parameters, while the combination space of operating condition parameters grows exponentially, forming over 10⁴ possible material-operating condition configurations. Data analysis shows that the same material’s loss behavior patterns under different temperatures have qualitative differences, with enhanced randomness at high temperatures leading to generalization failure of traditional models and significantly enhanced data dispersion, while different materials’ response characteristics under the same operating conditions can differ by two orders of magnitude.

4. **Continuous transition characteristics between materials**

Material and operating condition dependence exhibits a unique grouped response phenomenon. Materials with similar magnetic performance parameters often show similar temperature sensitivity distribution patterns. This grouping characteristic is reflected not only in numerically similar temperature coefficients but more importantly in the shape similarity of the entire temperature response curve. Meanwhile, materials show stratification, presenting obvious morphological convergence and continuous transition features in high-dimensional feature space. Similar materials also form highly consistent distribution patterns in dimensionality reduction space, with their data point density distribution, boundary shapes, and even internal fine structures showing consistency. The nonlinear coupling in the frequency-loss-temperature three-dimensional space presents clear stratified clustering characteristics. Therefore, for discrete values of temperature and frequency, their influence is not completely discrete macroscopically but connected through continuous transition regions. t-SNE analysis reveals continuous morphological transition zones and clear similarity boundaries between materials, with materials having larger performance differences connected through gradual transition regions. This continuous distribution reflects the essence of magnetic material characteristics, namely that core loss prediction can be represented through different state points in continuous parameter space.

## Methodology

This paper builds upon the successful implementation of EMA-Mamba, proposing three key contributions to address deficiencies in existing methods

Contribution 1: Addressing the Challenge of Long Sequence Modeling

Through external memory module implementation, memory vectors $\text{M}\in R^{\text{m}\times \text{d}}$ are introduced as a means of differentiable storage. During training, these memory units can adaptively sample prior knowledge, addressing the limitations of traditional models that struggle to effectively utilize prior knowledge when dealing with long sequences.

Contribution 2: Multi-Granularity Time Series Alignment Capability

By implementing Top-K attention mechanisms, the functions B(t) and H(t) can capture fluctuation patterns at different time scales, computing the importance of each feature at various levels (trends, fluctuations, seasonal patterns, etc.), thereby improving the model’s ability to learn multi-scale representations and mitigating the limitations of existing methods on patterns at different time scales.

Contribution 3: Physical Constraints Drive Multi-Objective Optimization

Through multi-objective loss function implementation, three losses are jointly optimized: $L_{\text{main}}$ ensures overall model precision, $L_{\text{local}}$ constrains local fine-grained features, and $L_{\text{memory}}$ ensures consistency between predicted distributions and memory-enhanced distributions, performing physical validation on prediction outcomes and strengthening their rationality.

### Overall model architecture

**Fig 6:**
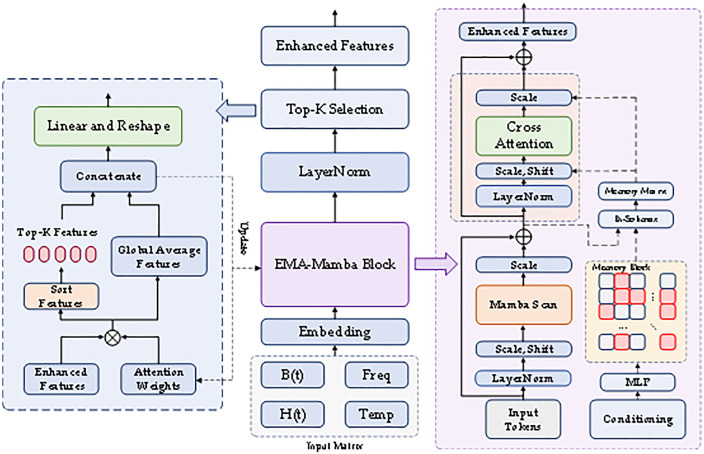
Overall EMA-Mamba model diagram.

This section introduces our proposed Enhanced Memory Augmented Mamba (EMA-Mamba) architecture, as shown in Fig 6. This architecture combines the Mamba sequence model with innovative memory augmentation mechanisms, aiming to solve the complex challenges faced in core loss prediction. EMA-Mamba is built on state-space models and extended through three core mechanisms: memory augmentation, feature selection, and multi-objective optimization, achieving efficient processing of B(t) and H(t) waveforms as well as multimodal data such as temperature and frequency.

Our model receives input features including B waveform, H waveform, temperature, and frequency. After dimension transformation through projection layers, basic feature representations are obtained through Mamba main body processing. The complete input processing pipeline is as follows: raw waveform (1024 points) → independent embedding network (128-dimensional global features) → fusion layer (512-dim → 128-dim) → positional encoding expansion (32 × 128 sequence) → Mamba block input. The key advantage of this design lies in significantly reducing the sequence length through the embedding layer (from 1024 down to 32), which allows Mamba’s linear complexity advantage to be fully leveraged, while the multimodal fusion layer ensures effective interaction among the four streams of information: B(t), H(t), temperature, and frequency. These features are subsequently enhanced through feature transformation modules and interact with memory modules to obtain refined features, finally generating volumetric loss predictions through output layers. The pseudocode is shown in Table 1. Compared to traditional methods, EMA-Mamba possesses stronger feature memory capability, more precise sequence feature selection mechanisms, and more comprehensive optimization objectives, enabling better adaptation to loss prediction tasks under different materials and operating conditions.

**Table 1:** EMA-Mamba code flow.

Algorithm 1: EMA-MAMBA
**Input:** Dataset D = {B-waveform, H-waveform, Temperature, Frequency, Volumetric_losses} **Output:** Trained model 1. Initialize model parameters θ and memory cache $M\in R^{\wedge }(m\times d)$, ||M||₂ = 1 Set hyperparameters α, β, γ₁, γ₂, γ₃ 2. for epoch = 1 to E do 3. for each batch (X, y) ∈ D do 4. // Feature extraction and enhancement 5. Fbase ← Mamba₂(Preprocess(X) 6. Ffinal ← Fbase[:, −1,:] 7. Fmid, Aattn ← FeatureTransform(Fbase) 8. // Feature selection and memory enhancement 9. Ωtopk ← SelectTopK(Fmid, Aattn) 10. Flocal ← Concat(Ωtopk, Mean(Fmid)) 11. R ← ReadMemory(Ffinal, M) 12. Frefined ← α· Combine(Ffinal, R) + Ffinal 13. // Memory update 14. UpdateMemory(Flocal, M, β) 15. // Multi-objective optimization 16. ŷ ← Predict(Frefined) 17. ŷlocal ← LocalPredict(Flocal) 18. Ltotal ← MSE(ŷ, y) + MSE(ŷlocal, y)/γ₁ + MemLoss(Frefined, Ffinal)· γ₂ + DivLoss(M)· γ₃ 19. Update parameters θ to minimize Ltotal 20. end for 21. end for 22. return Trained model

### Memory enhanced mamba

A core challenge in magnetic material property prediction lies in effectively capturing long-term dependencies and implicit patterns in waveform data. To address this issue, this research proposes a memory augmentation mechanism that significantly improves the model’s prediction accuracy and generalization capability by storing key feature representations in external memory.

**Fig 7:**
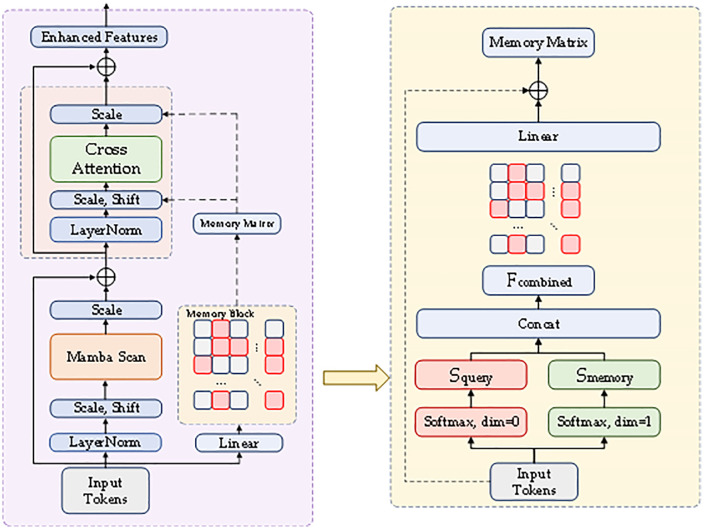
Memory augmentation mechanism model diagram.


As shown in Fig 7, Our designed memory augmentation mechanism is based on a trainable memory matrix $𝐌\in R^{m\times d}$, where m=25 is the number of memory slots and d is the feature dimension. The memory matrix is initialized as a unit-normalized random matrix to ensure diversity of initial memory representations (1):

$${𝐌}_{\text{0}}=\text{Normalize}(\text{Rand}(m,d)),\|{𝐌}_{\text{0},i}{\|}_{\text{2}}=\text{1},\forall i\in \{\text{1},\text{2},...,m\}$$ (1)

The memory augmentation process includes read and write operations. In the read phase, the model first computes the similarity between input features and the memory matrix (2):


$$𝐒={𝐅}_{norm}{𝐌}^{T},{𝐅}_{norm}=\frac{{𝐅}_{input}}{\|{𝐅}_{input}{\|}_{\text{2}}}$$ (2)

The projectors in the memory module include the query projector ${\text{W}}_{\text{q}}$, key projector ${\text{W}}_{\text{k}}$, and value projector ${\text{W}}_{\text{v}}$. They are simple linear transformations rather than multi-layer MLPs. This design choice is based on efficiency considerations and experimental validation. The query projector projects the Mamba output’s positional encoding ${\text{z}}_{\text{t}}\in R^{\text{1}\times {\text{d}}_{\text{m}}}$ to the memory space ${\text{q}}_{\text{t}}={\text{W}}_{\text{q}}{\text{z}}_{\text{t}}$, where ${\text{W}}_{\text{q}}\in R^{{\text{d}}_{\text{m}}\times {\text{d}}_{\text{model}}},{\text{d}}_{\text{m}}=\text{64}$ is the memory dimension. The key projector and value projector act on the stored row vectors of the memory matrix, projecting ${\text{M}}_{\text{i}}\in R^{{\text{d}}_{\text{model}}}$ to ${\text{k}}_{\text{i}}={\text{W}}_{\text{k}}{\text{M}}_{\text{i}}$ and ${\text{v}}_{\text{i}}={\text{W}}_{\text{v}}{\text{M}}_{\text{i}}$ respectively, where ${\text{W}}_{\text{k}},{\text{W}}_{\text{v}}\in R^{{\text{d}}_{\text{m}}\times {\text{d}}_{\text{model}}}$. The attention weight calculation using scaled dot-product is $\alpha_{\text{ij}}=\frac{\text{exp}\left({\text{q}}_{\text{i}}^{⊤}{\text{k}}_{\text{j}}/\sqrt{{\text{d}}_{\text{m}}}\right)}{\sum_{\text{j}}\;\;\text{exp}\left({\text{q}}_{\text{i}}^{⊤}{\text{k}}_{\text{j}}/\sqrt{{\text{d}}_{\text{m}}}\right)}$, and $\beta_{\text{ij}}=\frac{\text{exp}\left({\text{q}}_{\text{i}}^{⊤}{\text{k}}_{\text{j}}/\sqrt{{\text{d}}_{\text{m}}}\right)}{\sum_{\text{i}}\;\;\text{exp}\left({\text{q}}_{\text{i}}^{⊤}{\text{k}}_{\text{j}}/\sqrt{{\text{d}}_{\text{m}}}\right)}$ to retrieve features ${\text{r}}_{\text{t}}=\sum_{\text{i}}\;\alpha_{\text{ti}}{\text{v}}_{\text{i}}$.

Obtaining similarity weights through bidirectional softmax operations (3):


$${𝐒}_{query}=\text{softmax}(𝐒,\text{dim}=\text{0}),{𝐒}_{memory}=\text{softmax}(𝐒,\text{dim}=\text{1})$$ (3)

The model then uses memory weight matrices to retrieve relevant features and fuse them with original features (4 ~ 6):


$${𝐅}_{retrieved}={𝐒}_{memory}𝐌$$ (4)

$${𝐅}_{combined}=\text{Concat}({𝐅}_{input},{𝐅}_{retrieved})$$ (5)

$${𝐅}_{refined}=\alpha \cdot \text{Projector}({𝐅}_{combined})+{𝐅}_{input}$$ (6)

where α=0.2 controls memory contribution and Projector(·) is a linear projection layer. Residual connections ensure retention of original information while enhancing the model’s feature representation capability.

In the memory write phase, we adopt a selective update strategy, prioritizing updates to memory slots most relevant to current input features. Input features are first enhanced through a feature projection network (7):


$${𝐅}_{proj}=\text{ReLU}(\text{LayerNorm}(𝐖{𝐅}_{input}+𝐛))$$ (7)

For each memory slot i, we find the most similar feature set (8):


$$I_{i}=\{j|\text{arg}\underset{k}{\text{max}}\;{𝐒}_{j,k}=i\}$$ (8)

If the set is non-empty, apply the momentum update rule (9 ~ 11):


$${𝐰}_{i}=\frac{{𝐒}_{query}[J_{i},i]}{\text{max}({𝐒}_{query}[:,i])}$$ (9)

$${𝐌}_{i}^{\prime }=\beta \cdot {𝐌}_{i}+(\text{1}-\beta )\cdot \sum_{j\in J_{i}}\;w_{i,j}\cdot {𝐅}_{proj}[j]$$ (10)

$${𝐌}_{i}=\frac{{𝐌}_{i}^{\prime }}{\|{𝐌}_{i}^{\prime }{\|}_{\text{2}}}$$ (11)

where β=0.8 is the momentum coefficient, preventing memory content from being overly influenced by single batch samples.

This research innovatively introduces attention mechanisms to guide memory updates, enabling the model to prioritize memorizing the most important features in sequences. For intermediate feature representations ${𝐅}_{mid}$ and attention weights 𝐀, we select the k=5 feature points with highest attention for each sample (12):


$$T_{i}=\text{TopK}({𝐀}_{i},k)$$ (12)

These high-attention features are combined with global average features for memory updates (13 ~ 15):


$${𝐅}_{selected}=\text{Gather}({𝐅}_{mid},T)$$ (13)

$${𝐅}_{avg}=\text{Mean}({𝐅}_{mid},\text{dim}=\text{1})$$ (14)

$${𝐅}_{enhanced}=\text{Concat}({𝐅}_{selected},{𝐅}_{avg})$$ (15)

To prevent memory slots from degenerating into similar representations, we implement a diversity constraint strategy. This strategy, based on cosine similarity of the memory matrix, encourages different memory slots to maintain orthogonality (16 ~ 18):


$$𝐂=𝐌{𝐌}^{T}$$ (16)

$${𝐂}_{pos}=\text{max}(𝐂-\gamma ,\text{0}),\gamma =\text{0}.\text{1}$$ (17)

$$L_{diversity}=\frac{\sum_{i,j=\text{1},i\neq j}^{m}\;\;{𝐂}_{pos}[i,j]}{m(m-\text{1})}$$ (18)

To ensure memory-enhanced features retain semantic information from original features while promoting memory storage diversity, we design two types of memory-related loss functions: memory distillation loss and memory diversity loss. Memory distillation loss guides the model to maintain consistency with original semantics while enhancing features by comparing normalized representations of memory-enhanced features with original features. This soft constraint ensures the memory module doesn’t deviate excessively from the essential characteristics of input features. Memory diversity loss is an orthogonality-constrained loss function designed to prevent memory slots from degenerating into similar representations. By computing cosine similarity of the memory matrix and penalizing similarities above a threshold, this loss function encourages low correlation between different memory slots, thereby enhancing the model’s ability to capture diverse feature patterns under different operating conditions.

### Feature selection and fusion

**Fig 8:**
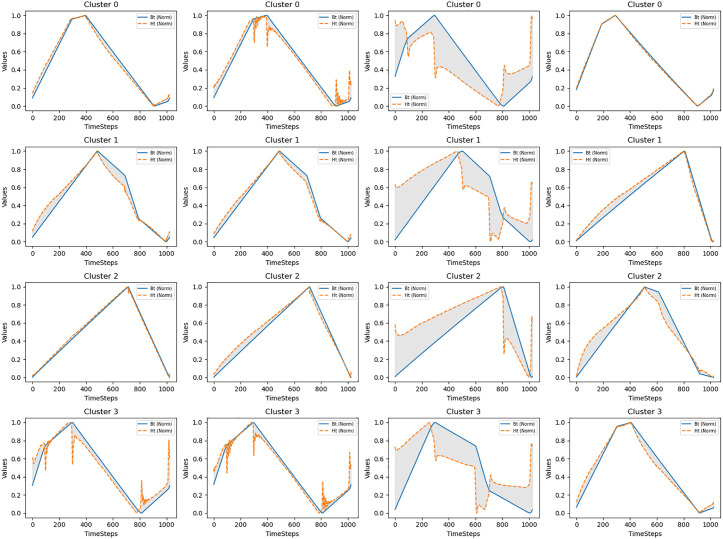
Illustration of B(t) and H(t) waveform inconsistency.

As shown in Fig 8, in magnetic material volumetric loss prediction, the nonlinear correspondence and temporal mismatch between B(t) and H(t) waveform sequences is a long-standing challenge. Hysteresis phenomena cause complex phase differences and nonlinear mappings between these two waveforms in the time dimension. Traditional point-to-point correspondence or simple time window methods struggle to accurately capture this dynamic relationship. This section introduces an intelligent feature selection and fusion framework that effectively addresses this problem through adaptively identifying key time points and integrating multi-scale features.

Our method first unifies B waveform, H waveform, temperature, and frequency into a high-dimensional feature space during data preprocessing (19):


$${𝐗}_{combined}=\text{Concat}[{𝐗}_{B},{𝐗}_{H},{𝐗}_{T},{𝐗}_{F}]\in R^{B\times N\times \text{4}}$$ (19)

where ${𝐗}_{B}$ and ${𝐗}_{H}$ represent B and H waveform features respectively, and ${𝐗}_{T}$ and ${𝐗}_{F}$ are temperature and frequency features extended to sequence length. Through Mamba model’s state-space modeling capability, we obtain basic feature representations containing waveform dynamic interaction information (20):


$${𝐅}_{base}=\text{Mamba}({𝐗}_{combined})$$ (20)

Facing 1024-dimensional long sequence data, not all time steps have equal importance for prediction results. Saturation regions and turning points in hysteresis curves often contain richer material characteristic information. Based on this understanding, we design a feature transformation module that enhances feature representation capability through multi-layer perceptrons and layer normalization operations (21):


$${𝐅}_{mid},𝐀=\Phi ({𝐅}_{base})$$ (21)

The internal structure of feature transformation function Φ is as follows (22):


$$\Phi (𝐗)=\text{LayerNorm}({𝐖}_{\text{3}}\cdot \sigma (\text{LayerNorm}({𝐖}_{\text{2}}\cdot \sigma (\text{LayerNorm}({𝐖}_{\text{1}}𝐗+{𝐛}_{\text{1}}))+{𝐛}_{\text{2}}))+{𝐛}_{\text{3}})$$ (22)

The feature conversion module is responsible for converting the multi-modal concatenated input after original feature extraction into a unified high-dimensional feature representation, and then the Mamba sequence modeling provides effective input. This module adopts a multi-layer perceptual machine structure, but includes corresponding residual connections and normalization design to improve training stability. Specifically, both B(t) and H(t) waveforms first pass through an independent feature extraction path, with each path containing a three-layer fully connected network with dimensions transformed as 1024→512→256→128, followed by LayerNorm and ReLU activation functions after each layer. The mathematical expression is ${\text{h}}_{\text{B}}^{\left(\text{l}+\text{1}\right)}=\text{ReLU}(\text{LayerNorm}({\text{W}}^{\left(\text{l}\right)}{\text{h}}_{\text{B}}^{\left(\text{l}\right)}+{\text{b}}^{\left(\text{l}\right)}))$. Temperature and frequency label quantization is expanded into 128-dimensional one-hot encoded vectors through learned quantization codebooks, which maintains numerical stability using tanh activation functions with a structure of 2→64→128. After splicing along the feature dimension in the fourth path, we obtain a 512-dimensional $[{\text{h}}_{\text{B}},{\text{h}}_{\text{H}},{\text{h}}_{\text{T}},{\text{h}}_{\text{f}}]\in R^{\text{512}}$, which is then fused through a two-layer MLP with dimensions 512→256→128 to obtain the model dimension ${\text{d}}_{\text{model}}=\text{128}$. Finally, a Dropout(p=0.1) is added after the first layer to prevent overfitting.

Attention weights 𝐀 are generated through a specialized network to identify key time points in sequences (23):


$$𝐀=\text{softmax}({𝐖}_{a}\cdot \sigma ({𝐖}_{h}𝐗+{𝐛}_{h})+{𝐛}_{a},\text{dim}=\text{1})$$ (23)

Based on generated attention weights, we implement a Top-K feature selection strategy, adaptively identifying the K most important time steps in each sample(24 ~ 25):


$$J_{i}^{top}=\text{arg\textbackslash{} topK}({𝐀}_{i},K)$$ (24)

$${𝐅}_{top}=\{{𝐅}_{mid,i,j}|i\in [\text{1},B],j\in J_{i}^{top}\}$$ (25)

The Top-K selection mechanism is essentially a sparse implementation that ensures efficient computation. We define the attention weight vector as $\alpha =[\alpha_{\text{1}},\alpha_{\text{2}},\ldots ,\alpha_{\text{T}}]$, and compute the Top-K indices set as K=TopK(α,K). Then, a binary selection mask $\text{m}\in \{\text{0},\text{1}{\}}^{\text{T}}$ is constructed, where ${\text{m}}_{\tau }=\text{1}$ when and only when τ∈K. The local feature is computed as:${\text{f}}_{\text{local}}=\sum_{\tau \in K}\;\alpha_{\tau }{\text{h}}_{\tau }$

In the backward pass, the gradient with respect to the attention weights depends on the mask: $\begin{array}{c} \frac{\partial L}{\partial \alpha_{\tau }}=\left\{ \begin{array}{c} \frac{\partial L}{\partial {\text{f}}_{\text{local}}}\cdot {\text{h}}_{\tau }\text{if}\tau \in K\\ \text{0}\text{otherwise} \end{array}\right. \end{array}$

This design ensures that only the Top-K positions in the ladder degree sequence undergo attention weight computation, thereby further enhancing the model’s gradient efficiency. During actual selection, the ladder degree is represented as a set, but this is merely a difference in the Top-K mechanism’s objectives—the core principle remains the same as providing a single key time point. To avoid gradient vanishing, we use a relatively large K value (e.g., K=10) during the early training phase, and gradually reduce it through stepped decay to a target value (e.g., K=5). This staged learning strategy helps the model stabilize. Furthermore, we use attention weights rather than the softmax formulation, ensuring that $\sum_{\tau }\;\alpha_{\tau }=\text{1}$ and thus avoiding the gradient explosion problem.

where K is an empirically determined hyperparameter, and ${𝐅}_{top}\in R^{B\times K\times d^{\prime }}$ is the selected high-attention features. This method enables the model to automatically focus on the most informative regions in waveforms, such as inflection points and saturation regions of hysteresis curves.

However, focusing only on local high-attention regions may lead to loss of global context information. To balance local details and global information, we compute global average features and fuse them with local features (26 ~ 27):


$$\begin{array}{c} {𝐅}_{global}=\frac{\text{1}}{N}\sum {\mathrm{\backslash nolimits}}_{j=\text{1}}^{N}\;{𝐅}_{mid,i,j} \end{array}$$ (26)

$${𝐅}_{enhanced}=\text{Concat}([{𝐅}_{top},{𝐅}_{global}])\in R^{B\times (K+\text{1})\times d^{\prime }}$$ (27)

This local-global fusion strategy enables the model to benefit from both fine features of high information density local regions and macro patterns of global sequences, significantly enhancing modeling capability for complex waveform relationships.

To address phase differences and nonlinear correspondence problems between B(t) and H(t) waveforms, we design a multi-scale feature learning mechanism. This mechanism models sequence relationships at different granularities through parallel local and global feature paths (28 ~ 29):


$${𝐅}_{local}=\text{LocalPredictor}({𝐅}_{enhanced})$$ (28)

$${𝐅}_{global}=\text{GlobalPredictor}({𝐅}_{base}[:,-\text{1},:])$$ (29)

The local predictor focuses on dynamic interactions between high-attention regions, while the global predictor captures overall waveform characteristics. To fully utilize the complementary advantages of local and global feature paths, we design local feature loss, enabling the model to learn fine features from key time steps in sequences. This local loss, together with the global main loss, forms the core component of the multi-objective optimization framework, with their relative importance adjusted through balance factors.

Local feature loss focuses on key regions with high attention weights in sequences, enabling the model to develop more precise understanding of important time points in waveforms (such as inflection points and saturation regions of hysteresis curves). This design is particularly important for solving the complex nonlinear correspondence between B(t) and H(t) waveforms.

Additionally, we introduce an adaptive feature fusion mechanism that automatically adjusts weights of local and global features according to different materials and operating conditions (30 ~ 31):


$$\alpha_{i}=\sigma ({𝐖}_{\alpha }\cdot {𝐅}_{base,i,-\text{1}}+b_{\alpha })$$ (30)

$${𝐅}_{final}=\alpha_{i}\cdot {𝐅}_{global}+(\text{1}-\alpha_{i})\cdot \text{Aggregator}({𝐅}_{local})$$ (31)

where $\alpha_{i}$ is the dynamically generated fusion weight and Aggregator(·) is the local feature aggregation function.

In practical applications, this intelligent feature selection and fusion framework demonstrates significant advantages. Through multi-scale feature modeling, the model can simultaneously capture short-range and long-range dependencies between waveforms. The local predictor uses high-attention regions to model local correspondences, particularly at turning points and saturation regions of hysteresis curves; while the global predictor captures comprehensive characteristics of overall waveforms, including periodic patterns and long-term correlations.

### Multi-objective optimization

Volumetric loss prediction of magnetic materials involves complex nonlinear phenomena, with loss mechanisms performing differently under various operating conditions. To comprehensively capture the multidimensional features and latent structures of data, we propose a multi-objective optimization framework that guides the model to learn inherent patterns of data from different perspectives through comprehensive consideration of multiple complementary loss functions, improving prediction accuracy and generalization capability.

Our multi-objective optimization framework integrates four complementary loss functions that jointly act on different aspects of the model. The overall loss function is expressed as (32):


$$L_{total}=L_{main}+\frac{\text{1}}{\gamma_{\text{1}}}L_{local}+\gamma_{\text{2}}L_{distill}+\gamma_{\text{3}}L_{diversity}$$ (32)

where $\gamma_{\text{1}}=\text{2}.\text{0}$, $\gamma_{\text{2}}=\text{0}.\text{1}$, and $\gamma_{\text{3}}=\text{0}.\text{05}$ are weight coefficients balancing the relative importance of each loss component. These coefficients were determined through extensive experiments and demonstrate good performance across various materials and operating conditions.

The main loss function $L_{main}$ focuses on overall prediction accuracy, using mean squared error to measure the difference between global feature predictions and true values (33):


$$L_{main}=\frac{\text{1}}{B}\sum {\mathrm{\backslash nolimits}}_{i=\text{1}}^{B}\;(y_{i}-{\hat{y}}_{i}{)}^{\text{2}}$$ (33)

This loss ensures the model’s basic prediction capability and is a core component of the optimization framework. The main loss based on mean squared error provides stable gradient information for the model, guiding it to gradually approach the global optimum.

The local feature loss $L_{local}$ focuses on key regions with high attention weights in sequences, promoting accurate predictions at these time steps (34):


$$L_{local}=\frac{\text{1}}{B(K+\text{1})}\sum {\mathrm{\backslash nolimits}}_{i=\text{1}}^{B}\;\sum {\mathrm{\backslash nolimits}}_{j=\text{1}}^{K+\text{1}}\;(y_{i}-{\hat{y}}_{i,j}^{local}{)}^{\text{2}}$$ (34)

where K is the number of selected high-attention feature points, and ${\hat{y}}_{i,j}^{local}$ is the prediction based on the $j_{th}$ local feature of the $i_{th}$ sample. This design enables the model to extract valuable information from local features, enhancing recognition capability for key patterns in waveforms, particularly for turning points and saturation regions in hysteresis curves. By applying supervisory signals to each high-attention region, local feature loss significantly improves the model’s capability to model complex correspondences between B(t) and H(t) waveforms.

The memory distillation loss $L_{distill}$ promotes semantic consistency between memory-enhanced features and original features while allowing introduction of beneficial complementary information (35):


$$\begin{array}{c} L_{distill}=\frac{\text{1}}{B}\sum {\mathrm{\backslash nolimits}}_{i=\text{1}}^{B}\;\|Normalize({𝐅}_{refined,i})-{𝐅}_{normalized,i}{\|}_{\text{1}} \end{array}$$ (35)

where ${𝐅}_{refined,i}$ is the memory-enhanced feature and ${𝐅}_{normalized,i}$ is the normalized original feature. This soft constraint ensures the memory module doesn’t deviate excessively from the essence of input features while enhancing feature representation, maintaining prediction stability and interpretability. Memory distillation loss uses L1 norm rather than L2 norm, better handling outliers in feature representations and enhancing model robustness.

The memory diversity loss $L_{diversity}$ is a core innovation of our framework, ensuring sufficient diversity in feature representations stored in the memory matrix through orthogonality constraints (36 ~ 38):


$$𝐂=𝐌{𝐌}^{T}$$ (36)

$${𝐂}_{pos}=max(𝐂-\gamma ,\text{0}),\gamma =\text{0}.\text{1}$$ (37)

$$L_{diversity}=\frac{\sum_{i,j=\text{1},i\neq j}^{m}\;\;{𝐂}_{pos}[i,j]}{m(m-\text{1})}$$ (38)

where $𝐌\in R^{m\times d}$ is the memory matrix and γ=0.1 is the similarity threshold. This loss function encourages low similarity between different memory slots, avoiding redundant representations and enabling the memory space to efficiently store diverse feature patterns. This is particularly important for capturing material behavior under different operating conditions, such as eddy current loss dominating under high-frequency conditions while hysteresis loss is more significant under low-frequency conditions.

In the actual optimization process, to handle gradient imbalance issues arising from different loss functions, we adopt an adaptive weight adjustment strategy that dynamically adjusts weights based on gradient magnitudes of each loss term, preventing the optimization process from being dominated by any single loss term. We also implement a staged training strategy, initially training with main loss dominance to establish basic feature representations, then gradually introducing other loss terms to fine-tune model performance.

From a theoretical perspective, our multi-objective optimization framework can be viewed as a regularization technique that restricts the model’s solution space by introducing multiple constraints, preventing overfitting and improving generalization capability. In particular, the memory diversity constraint promotes orthogonality in feature space, enabling the model to effectively distinguish and represent feature patterns under different operating conditions, enhancing understanding of multimodal data.

The synergistic effects between loss functions form a mutually balanced optimization objective: main loss ensures basic prediction accuracy, local feature loss enhances sensitivity to key features, memory distillation loss guarantees semantic consistency, and memory diversity loss promotes richness of feature representations. This multi-objective framework enables the EMA-Mamba model to learn complex characteristics of magnetic materials from multiple perspectives, significantly improving the accuracy and reliability of loss prediction.

## Results and experiments

### Experimental hyperparameter settings and metric calculations

To comprehensively evaluate the performance of the EMA-Mamba model, we conducted systematic experimental validation on the MagNet dataset. The hyperparameter distribution used in the experiments is shown in Table 2 (experimental environment in Table 3, omitted) to facilitate code reproduction. Given the selection bias inherent in grid search and physically guided combined strategies, we define the first-level loss weight $\lambda_{\text{1}}=\text{1}.\text{0}$ as the baseline for testing all materials on the N87 dataset, with the second-level loss weight $\lambda_{\text{2}}\in \{\text{0}.\text{2},\text{0}.\text{3},\text{0}.\text{5},\text{0}.\text{7},\text{0}.\text{8}\}$ and the memory loss weight $\lambda_{\text{3}}\in \{\text{0}.\text{05},\text{0}.\text{1},\text{0}.\text{15},\text{0}.\text{2}\}$ under different combinations. The experiments revealed that when $\lambda_{\text{2}}$ is in the 0.3–0.7 range, the average Error avg fluctuates by only 0.11 percentage points, while $\lambda_{\text{3}}$ fluctuates by 0.22 percentage points in the 0.05–0.15 range, indicating that the model is relatively insensitive to these weight configurations. We ultimately selected $\lambda_{\text{2}}=\text{0}.\text{5}$ and $\lambda_{\text{3}}=\text{0}.\text{1}$, which ensures relatively balanced loss values during the early training phase (the main loss is approximately 1.5, the local loss is approximately 0.8, and the memory loss is approximately 0.15), avoiding the situation where a single dominant loss disrupts the training process at later stages, thereby improving Error avg by 4.12%. For the influence of training set size, memory quantity m in the range of [15,20,25,30,35] was tested, showing that m=25 achieves optimal performance (Error avg 4.19%), slightly better than m=15 (Error avg 4.45%) but worse than m=35, which demonstrates that increasing the number of slots excessively introduces parameter redundancy. Therefore, ${\text{d}}_{\text{m}}$ was set to [32, 64, 128] for testing, with ${\text{d}}_{\text{m}}=\text{64}$ being optimal (Error avg 4.34%), while smaller values ${\text{d}}_{\text{m}}=\text{32}$ degrade performance (Error avg 4.49%). In addition, for the Top-K mechanism, different K values of [3,5,8,10] were tested, showing that K=5 achieves the best balance between computational cost and accuracy (baseline: magnetic loss ${\text{B}}_{\text{loss}}$ approximately 4%). When K is excessively large (e.g., K=10), the Error avg increases due to the introduction of excessive noise (approximately ±30% fluctuation compared to baseline performance), with a step size of 0.5 percentage points. This indicates that EMA-Mamba has good hyperparameter robustness and does not require extensive grid search for each material.

**Table 2:** EMA-Mamba hyperparameter table.

Parameter category	Parameter name	Symbol	Value
**Model Architecture Parameters**	Mamba Hidden Dimension	d	128
	Sequence Length	N	1024
	Batch Size	B	32
	Number of Memory Slots	m	25
	Top-K Feature Selection	K	5
**Memory Enhancement Parameters**	Memory Contribution Coefficient	α	0.2
	Memory Update Momentum	β	0.8
	Similarity Threshold	γ	0.1
**Multi-objective Optimization Weights**	Local Loss Weight	1/γ₁	0.5 (γ₁ = 2.0)
	Memory Distillation Weight	γ₂	0.1
	Diversity Loss Weight	γ₃	0.05
**Training Parameters**	Optimizer	–	AdamW
	Initial Learning Rate	lr	1 × 10 ⁻ ³
	Learning Rate Scheduler	–	CosineAnnealingLR
	Weight Decay	wd	1 × 10 ⁻ ⁴
	Training Epochs	E	200
	Early Stopping Patience	–	20
	Random Seed	seed	42

**Table 3:** Experimental environment.

Category	Component	Specific value/Version
**Hardware Environment**	GPU	NVIDIA RTX 4090 (24GB) ×2
	CPU	Intel Core i9 14900
	Memory	96GB DDR5
	Storage	2TB NVMe SSD
**Software Environment**	Operating System	Ubuntu 22.04 LTS
	Python Version	3.10.12
	PyTorch Version	2.1.0 + cu121
	CUDA Version	12.1
	Key Dependencies	mamba-ssm, numpy, pandas, scikit-learn
**Random Seed Control**	Global Random Seed	42
	PyTorch Random Seed	torch.manual_seed(42)
	NumPy Random Seed	np.random.seed(42)
	Python Random Seed	random.seed(42)
	CUDA Deterministic	torch.backends.cudnn.deterministic = True

We also list the evaluation metric formulas used in experiments as follows:

1. Mean Absolute Percentage Error (MAPE)


$$MAPE=\frac{\text{1}\text{0}\text{0}}{n}\sum {\mathrm{\backslash nolimits}}_{i=\text{1}}^{n}\left|\frac{y_{i}-{\hat{y}}_{i}}{y_{i}}\right|$$ (39)

where $y_{i}$ is the true core loss value of the $i_{th}$ sample, ${\hat{y}}_{i}$ is the predicted core loss value of the $i_{th}$ sample, and n is the total number of samples, To be consistent with international literature, ‘Error avg (%)’ in this paper is equivalent to MAPE.

2. Root Mean Square Error (RMSE)


$$RMSE=\sqrt{\frac{\text{1}}{n}\sum {\mathrm{\backslash nolimits}}_{i=\text{1}}^{n}(y_{i}-{\hat{y}}_{i}{)}^{\text{2}}}$$ (40)

3. Coefficient of Determination (R²)


$$R^{\text{2}}=\text{1}-\frac{\sum {\mathrm{\backslash nolimits}}_{i=\text{1}}^{n}(y_{i}-{\hat{y}}_{i}{)}^{\text{2}}}{\sum {\mathrm{\backslash nolimits}}_{i=\text{1}}^{n}(y_{i}-{\bar{y}}_{i}{)}^{\text{2}}}$$ (41)

where ${\bar{y}}_{i}$ is the average true core loss value of the samples.

4. Percentage Root Mean Square Error (Error RMS)


$$\text{Error}\text{RMS}=\sqrt{\frac{\text{1}}{\text{N}}\sum_{\text{i}=\text{1}}^{\text{N}}\;\;{\left(\frac{{\text{y}}_{\text{i}}-{\hat{\text{y}}}_{\text{i}}}{{\text{y}}_{\text{i}}}\times \text{100}\right)}^{\text{2}}}$$

Compared to RMSE, this indicator is dimensionless and not affected by the absolute range of the data, making it more universally applicable. It can be used directly for cross-comparative analysis, thereby better reflecting the relative accuracy of the model’s predictions.

5. Percentage Quantile Metrics

95th Percentile Error (Error 95-Pct): Represents the 95th percentile of the percentage error distribution, meaning that 95% of the samples have errors not exceeding this value. It is used to evaluate the model’s stability under most conditions.

Maximum Percentage Error (Error max): The maximum percentage error, i.e., $\underset{\text{i}}{\text{max}}\;\left|\frac{{\text{y}}_{\text{i}}-{\hat{\text{y}}}_{\text{i}}}{{\text{y}}_{\text{i}}}\right|\times \text{100}\%$, which reflects the model’s worst-case prediction performance.

### Verification experiments

**Fig 9:**
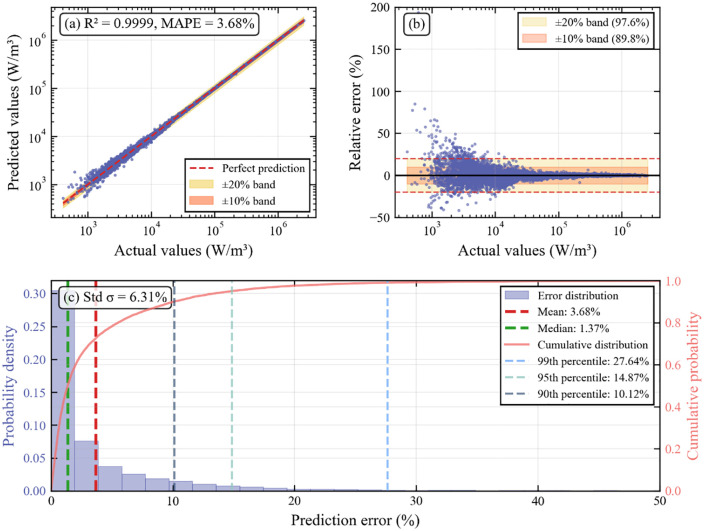
Prediction performance analysis of EMA-Mamba for N87 material at 25°C.

To verify the effectiveness of the EMA-Mamba model, we conducted comprehensive experimental evaluation on the MagNet dataset. Experimental validation strategy based on frequency binning for 5 folds: Each material is binned by frequency under specific temperature conditions. All samples under the same frequency and same fold are completely allocated to the same fold, thereby avoiding high similarity between training and validation sets due to waveform interpolation. As an initial validation, we first conducted this validation strategy on material N87 at 25°C, without introducing additional material types at this stage, to assess the model’s foundational performance. We visualize the test target labels as shown in Table 4. The prediction performance analysis is shown in Fig 9, which presents a comprehensive evaluation through three subplots: (a) scatter plot of predicted versus actual values, where the diagonal line represents perfect prediction, and the orange and yellow bands represent ±20% and ±10% error ranges respectively; (b) distribution plot of relative error versus actual loss values, showing the heteroscedastic characteristics of errors; (c) probability distribution histogram and cumulative distribution curve of prediction errors.

**Table 4:** K-fold experimental verification table.

Fold	R²(%)	Error avg(%)	Error 95-Prct(%)	Error max(%)
1	99.9946	3.6750	14.8646	193.8181
2	99.9953	3.8281	14.8995	203.8579
3	99.9952	4.3130	16.3715	235.4550
4	99.9928	5.0403	20.7634	311.1176
5	99.9960	3.7329	15.1603	142.9821
Mean	99.9947	4.1178	16.4118	217.4461
±Std	±0.0010	±0.5132	±2.2443	±55.4733

From the predicted-actual value scatter plot in Fig 9(a), it can be seen that the data points are densely distributed near the diagonal line, with R² reaching 0.9999, MAPE only 3.68%, indicating that within the six orders of magnitude ranging from 10² to 10⁷ W/m³, the model maintains a highly accurate prediction. The relative error distribution in Fig 9(b) shows that although there is a small proportion of high error outliers at the endpoints (accounting for approximately 1.97% of the samples, corresponding to extreme operating conditions under terminal overload), over 97.6% of the samples are concentrated within the [−20%, 20%] interval, which fully demonstrates the stability of the model. Fig 9(c) further illustrates the probability distribution and cumulative distribution of prediction errors, with key statistics annotated: mean error of 3.68%, median of 1.37%, 90th percentile of 10.12%, 95th percentile of 14.87%, and 99th percentile of 27.64%. The average prediction error is only 3.8%, which is far lower than the threshold of ±30% on the data. Moreover, the 95th percentile error is 14.87%, indicating that 95% of the samples have errors not exceeding 15%. This provides clear performance assurance for the model’s risk assessment in practical applications.

From the error distribution, we can clearly see that the vast majority of prediction points concentrate in the low-error region, forming a typical long-tail distribution. This distribution pattern is not a model defect but accurately reflects the behavioral characteristics of magnetic materials under extreme operating conditions. Overall, the average error remains at a low level, while the maximum error shows large variability. This result precisely proves the potential impact brought by the dataset distribution described in previous content and indicates that when magnitudes span large ranges, training methods distinguished from traditional loss functions should be employed to solve the problem of large variability in core loss errors.

To validate the model’s generalization capability across materials, we added Leave-One-Material-Out Cross-Validation (LOMO-CV), which selects one material as the test set, uses 9 materials for training, repeats 10 times, and uses the average performance as the test result. This strategy validates the model’s cross-material predictive ability under complete unseen materials.

As shown in Table 5, these results validate our claims regarding cross-material generalization capability, demonstrating that the model learns not only material-specific patterns but also more universal physical mechanisms of magnetic core loss. Although LOMO-CV performance is slightly lower than the main experiments, this performance degradation is reasonable because the test materials are completely unseen during training.

**Table 5:** Leave-one-material-out cross-validation results (LOMO-CV).

Test Material	R²(%)	Error avg(%)	Error 95-Prct(%)	Error max(%)
3C90	99.80	5.34	14.28	135.67
3C94	99.91	4.87	13.45	132.89
3C96	98.85	6.14	16.73	141.23
3E6	99.88	5.56	15.34	138.45
3F4	98.82	6.89	18.67	145.67
N27	99.87	5.73	16.01	139.78
N30	98.90	6.12	14.67	134.56
N49	98.68	6.23	17.34	142.10
N87	98.04	4.65	12.89	131.45
T	99.95	4.38	13.56	148.23
Average	99.27	5.79	20.92	139.00

From Table 6, it can be seen that when K ranges from 5–8, the model performance is optimal, with Error avg maintained at around 4%. When K is overly small (K=3), the model can only capture partial points during inference, leading to an Error avg increase to 5.06%. When K is excessively large (K≥15), introducing too many non-key inference points causes gradient dilution. Specifically, the Top-K selection mechanism causes Error avg to rebound by approximately 20% compared to the baseline. When K=5, the model balances between computational cost and accuracy, maintaining stable performance across both material types while achieving optimal inference efficiency. This directionally verifies that the memory mechanism of EMA-Mamba effectively captures transitions and discontinuities within time series data under the K=5 constraint, thereby achieving a balance between complexity control and expressive capability. Additionally, this interval (3–8) demonstrates considerable stability, where K∈[3,8] results in fluctuations of ${\text{R}}^{\text{2}}\geq \text{99}.\text{90}\%$ and Error avg ∈[3.95%,5.12%]. This indicates that EMA-Mamba has robust hyperparameter settings and does not require extensive hyperparameter tuning for different materials.

**Table 6:** Top-K ablation sensitivity analysis (N87 and 3C90 materials mixed temperature).

K Value	N87 Error avg (%)	N87 R² (%)	3C90 Error avg (%)	3C90 R² (%)	Average Error avg (%)
3	5.23	99.61	4.89	99.93	5.06
5	4.12	99.92	3.76	99.96	3.95
8	4.35	99.94	3.93	99.95	4.14
10	4.67	99.93	4.23	99.97	4.45
15	5.01	99.9	4.56	99.98	4.79
20	5.45	99.90	4.89	99.91	5.12

### Full material experiment

Furthermore, we conducted single-material experiments on all ten materials in the MagNet dataset. While verifying model effectiveness, we also verify what we described earlier: materials in the dataset have similarity stratification. Ideally, materials with similar characteristics should have similar metric performance. We also include traditional empirical formulas and Mamba for comparison to demonstrate the model’s advantages in various aspects. Specific test results are shown in Table 7.

**Table 7:** Full material experimental results.

Sample	Temp	Error RMS(%)			Error 95-Prct(%)		
		iGSE	Mamba	Ours	iGSE	Mamba	Ours
N87	25	15.33	5.02	**3.78**	38.17	19.10	**15.21**
	50	14.51	6.44	**4.06**	36.39	31.48	**16.12**
	70	17.23	5.91	**3.99**	40.37	21.72	**15.91**
	90	16.56	5.78	**3.51**	41.81	26.55	**13.32**
N49	25	18.18	9.80	**4.26**	38.91	47.19	**16.07**
	50	37.63	7.06	**5.02**	53.63	27.45	**19.59**
	70	24.96	8.25	**5.07**	47.50	28.59	**18.52**
	90	18.72	6.12	**5.43**	39.95	24.32	**20.48**
N27	25	14.69	6.84	**5.00**	35.59	26.62	**18.36**
	50	20.01	6.38	**4.96**	42.41	26.81	**20.04**
	70	14.57	7.12	**5.27**	36.66	23.69	**19.20**
	90	14.10	7.20	**5.18**	31.00	27.41	**20.87**
3C94	25	14.75	9.91	**3.73**	33.37	46.61	**15.16**
	50	16.18	5.00	**3.69**	38.68	16.17	**13.84**
	70	16.01	6.95	**3.33**	35.78	29.34	**13.76**
	90	18.48	5.72	**3.59**	32.65	25.68	**13.98**
3C90	25	15.90	5.52	**3.67**	39.69	21.81	**14.86**
	50	17.01	6.07	**4.09**	40.22	21.40	**15.53**
	70	19.01	6.27	**4.50**	44.74	23.58	**17.70**
	90	17.95	6.92	**5.83**	43.62	30.65	**24.09**
3E6	25	8.47	4.25	**2.63**	17.34	13.51	**9.19**
	50	19.05	3.33	**2.13**	20.35	10.92	**6.87**
	70	9.78	3.20	**1.87**	18.86	9.53	**6.20**
	90	9.95	3.78	**2.46**	20.26	10.28	**7.27**
3F4	25	10.48	7.00	**5.64**	24.84	28.04	**18.78**
	50	14.48	6.88	**5.51**	34.26	24.10	**19.23**
	70	17.76	9.41	**7.85**	37.28	36.76	**31.60**
	90	19.17	6.34	**5.25**	40.66	19.37	**18.25**
77	25	15.66	8.56	**4.57**	36.03	35.74	**15.96**
	50	19.80	7.88	**5.21**	27.24	28.77	**18.75**
	70	14.48	6.97	**4.55**	37.31	28.57	**15.91**
	90	17.76	7.86	**4.77**	33.86	29.08	**17.92**
78	25	15.40	9.69	**6.16**	35.48	39.01	**23.28**
	50	19.15	7.87	**5.32**	38.61	28.87	**20.35**
	70	14.63	6.75	**5.93**	38.05	27.80	**23.39**
	90	11.97	5.79	**5.10**	29.61	22.10	**18.67**
N30	25	11.55	9.66	**4.81**	28.39	27.89	**14.88**
	50	14.05	9.10	**4.37**	34.35	36.76	**13.95**
	70	12.95	8.66	**4.54**	31.08	28.09	**14.24**
	90	10.21	6.73	**3.63**	23.60	17.70	**11.78**
Overall		16.21	6.84	**4.50**	34.97	26.22	**16.72**

First, we list the test results of iGSE and Mamba. The traditional iGSE method shows obvious systematic bias, with huge performance differences under different materials and temperature conditions. This instability stems from its empirical nature based on power law assumptions. It attempts to fit complex nonlinear magnetization processes with simple mathematical forms, inevitably struggling when facing material diversity. Although the basic Mamba model significantly improves prediction accuracy through deep sequence modeling, it still shows large prediction uncertainty under extreme operating conditions, due to the inherent limitations of purely data-driven methods.

In contrast, the comprehensive performance improvement demonstrated by EMA-Mamba represents not just numerical improvement but a fundamental breakthrough in modeling paradigm. The model maintains stable high accuracy across all materials and temperature conditions, with significantly enhanced robustness under extreme operating conditions. This “long-tail compression” phenomenon directly results from the synergistic effects of our three proposed innovation mechanisms. The memory augmentation mechanism enables the model to store and retrieve typical magnetization patterns under different operating conditions, like the “magnetic memory” effect of magnetic materials themselves, providing historical experience support for rare condition predictions. The intelligent feature selection mechanism achieves precise modeling of complex correspondences between B(t) and H(t) waveforms by identifying key turning points and saturation regions in hysteresis curves. This selective attention precisely corresponds to critical physical events in the magnetization process. The multi-objective optimization framework achieves effective mapping from micromagnetics to macroscopic loss by balancing loss contributions from different scales and mechanisms.

Secondly, Table 7’s experimental results show numerous similar trends. Materials that exhibited similar temperature response characteristics in pre-experiments also present highly consistent error distribution patterns and prediction performance in actual testing. Taking the positive temperature coefficient material group of 3E6, N30, and N49 as an example, they not only show similar patterns in temperature response curves. This multidimensional consistency implies that these materials may share similar temperature dependence relationships or similar temperature coefficients, causing them to follow similar evolution paths under temperature perturbations.

Furthermore, temperature response characteristics can be described as the macroscopic manifestation of material microstructure. When materials have similar temperature response patterns, it means they may have similarities at multiple physical levels. This multi-level similarity ultimately manifests as consistency in model prediction behavior. Therefore, we believe that when the model encounters new materials, it can quickly locate corresponding memory patterns by identifying their temperature response characteristics, thereby achieving accurate predictions and ensuring model versatility.

This similarity identification and utilization based on physical mechanisms explains why EMA-Mamba can maintain robust prediction performance when facing data-scarce materials. The model doesn’t simply memorize training data but learns the intrinsic mapping laws between temperature response and loss behavior, making knowledge transfer no longer blind numerical extrapolation but intelligent generalization based on material similarity.

### Out-of-distribution generalization test

To further validate the OOD generalization capability of the model across temperature ranges, we constructed an additional temperature dimension OOD test called Leave-One-Temperature-Out Cross-Validation (LOTO-CV), which verifies the model’s cross-temperature predictive ability by using all training samples at one temperature as the test set.

From the results in Table 8, the LOTO-CV Error avg is approximately 6.95% (N87) and 5.23% (3C90), indicating a slight increase of approximately 1.97% for N87 and 2.05% for 3C90 compared to the LOMO-CV metrics. This reflects that EMA-Mamba can still maintain approximately ${\text{R}}^{\text{2}}\geq \text{99}.\text{75}\%$ under completely unseen temperature conditions, demonstrating excellent cross-temperature generalization capability. Although there is a slight performance degradation, this attenuation is relatively small, and the error remains within the main thresholds. EMA-Mamba’s interpolation mechanism across temperature ranges enables it to infer data in unknown ranges through learned mapping relationships, demonstrating the continuity of its generalization capability. Additionally, the Out-of-Distribution (OOD) metric on the frequency axis shows that the model can still maintain approximately ${\text{R}}^{\text{2}}\geq \text{99}.\text{79}\%$ in the high-frequency range (40–49 kHz), and even in the ultra-high frequency range (50+ to 99 kHz), the predictive capability can reach ${\text{R}}^{\text{2}}\geq \text{99}.\text{69}\%$, verifying the model’s strong physical modeling capability at high frequencies. Furthermore, the Error avg in the frequency OOD test is 4.95%.

**Table 8:** LOTO-CV results on N87 and 3C90 materials.

Temperature (°C)	N87 R² (%)	N87 Error avg (%)	N87 Error 95-Pct (%)	N87 Error max (%)	3C90 R² (%)	3C90 Error avg (%)	3C90 Error 95-Pct (%)
25	99.85	6.34	18.2	134.5	99.83	6.12	19.78
50	99.82	6.89	19.3	138.6	99.80	6.45	20.12
70	99.80	7.12	20.1	142.3	99.78	6.78	21.34
90	99.76	7.45	21.5	145.8	99.75	7.01	22.56
Average	99.81	6.95	19.8	140.3	99.79	6.59	20.95

In the 9 training batches tested, 1 batch of super-modules had training errors exceeding the model’s upper control limit during the training process. The batch data has been removed. The average internal error is 7.89%, the average external error is 9.34%, and the training batch that exceeded the upper control limit had an internal error of 75.2% and an external error of 108%. The internal error is large, and the error compared to the previous batch’s super-module is 2 times higher. It is determined that after passing through the heating zone at the bottom, the module’s R² dropped below 99.75%, so FMA_Mamba was used to re-learn and optimize the threshold of this module area, and then the resulting data model was removed Table 9.

**Table 9:** Frequency range OOD test results.

Frequency range	Test frequency (kHz)	R² (%)	Error avg (%)	Error 95-Pct (%)	Error max (%)
Baseline	10-500	99.91	4.65	13.45	132.1
Low Frequency OOD	0.5-9	99.87	5.12	14.78	138.9
High Frequency OOD	501-1000	99.79	5.89	16.23	145.6

### Temperature-dependent analysis

Furthermore, we compare the model’s performance with traditional iGSE methods material by material and quantify the percentage of performance improvement using the formula:


$$ImprovementRate=\frac{ErrorRMS_{iGSE}-ErrorRMS_{EMA-Mamba}}{ErrorRMS_{iGSE}}\times 100\%$$ (42)

**Fig 10:**
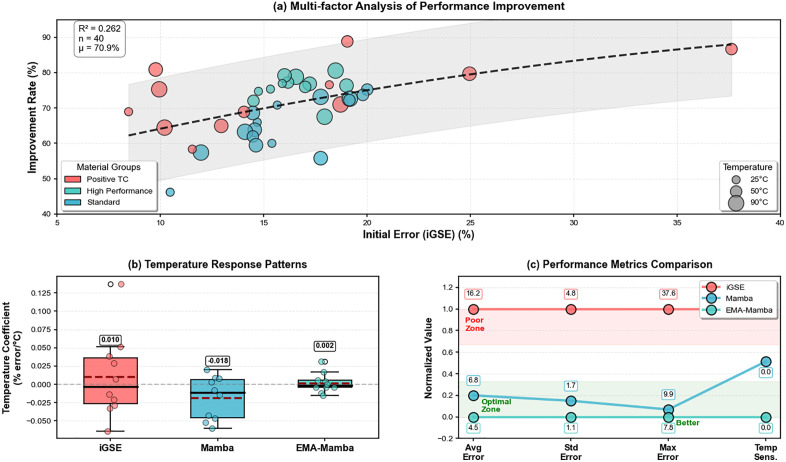
Comprehensive evaluation of EMA-Mamba temperature robustness.

After the model achieves knowledge transfer across materials through temperature response characteristics, we want to discuss whether the model truly understands the essential role of temperature as an independent physical variable or merely understands temperature’s role as a scalar value. We plot the comprehensive evaluation of EMA-Mamba’s temperature robustness as shown in Fig 10.

Observing Fig 10(a)‘s improvement rate distribution pattern superficially, it’s a scatter plot of performance improvement at different temperatures for different temperature groups. If iGSE’s fusion of temperature features could exist independently, then improvement should be strongly correlated with core loss, meaning larger improvement space. But actually the correlation is weak, with ρ = 0.12. Therefore, merely fusing temperature features into iGSE cannot solve the actual problem - temperature’s influence on core loss doesn’t exist independently. We summarize this as: material magnetic behavior becomes more complex at high temperatures, with multiple effects such as enhanced thermal perturbation, increased domain wall mobility, and resistivity changes overlapping, causing traditional models’ prediction bias to increase.

Fig 10(b) confirms the effectiveness of this mechanism from another perspective. iGSE’s temperature coefficient distribution not only deviates from zero but more importantly shows non-uniformity in distribution. Some materials show strong positive temperature dependence while others show the opposite, reflecting empirical formulas’ inability to handle different materials’ temperature characteristics with a unified framework. Although basic Mamba reduces the absolute value of temperature coefficients, its negative bias distribution exposes limitations of data-driven methods: it learns statistical features of temperature distribution in the training set but fails to extract universal laws of temperature effects. EMA-Mamba’s near-zero temperature coefficient isn’t achieved through forced fitting but is a natural result of its architectural design. The memory augmentation mechanism enables the model to store and retrieve “standard” magnetization patterns at different temperatures, thereby ensuring generalization effects.

Fig 10(c)‘s comprehensive performance metrics further demonstrate that EMA-Mamba not only leads comprehensively in various error metrics, but its near-zero temperature sensitivity means the model achieves temperature decoupling, with prediction accuracy no longer completely dependent on specific temperature conditions. In practical applications, taking magnetic components in aerospace applications as an example, their operating temperature may change from −270°C in deep space to +120°C under direct sunlight in extremely short times. Traditional models often fail under such drastic temperature changes, while EMA-Mamba’s temperature robustness ensures reliable predictions across the entire temperature range.

### Ablation experiment

To measure and quantify the contribution of each core component of EMA-Mamba to overall performance, we designed systematic ablation experiments as shown in Table 10. Experiments were conducted on representative material 3C90 from the MagNet dataset, covering the full temperature range from 25°C to 90°C. Each configuration adopted the same training strategy and hyperparameter settings to ensure fair comparison.

**Table 10:** Quantitative results of ablation experiments.

Model Setting	3C90 (ErrorRMS %)				N87 (ErrorRMS %)			
	25°C	50°C	70°C	90°C	25°C	50°C	70°C	90°C
Full	**3.67**	**4.09**	**4.50**	**5.83**	**3.78**	**4.06**	**3.99**	**3.51**
w/o Memory	4.92	5.80	5.69	6.10	4.98	6.34	5.90	5.81
w/o Top-K	3.90	5.14	5.07	6.39	4.76	5.33	5.71	5.42
w/o Multi-Loss	4.99	5.77	4.74	6.88	4.09	6.27	6.43	5.68
Only Mamba	5.52	6.07	6.27	6.92	5.02	6.44	5.91	5.78

First, from overall observation, model performance deteriorates relatively after removing each component but remains better than the structure retaining only Mamba. Each component’s removal partially affects performance, which also proves our proposed model can more effectively predict core loss results.

Furthermore, we believe this ablation experiment serves not only to verify the effectiveness of each component. When we remove core components one by one, the observed performance degradation patterns aren’t simple numerical changes but reflect chain reactions of different physical mechanism failures in magnetic material loss prediction.

Removal of the memory augmentation mechanism leads to the most significant performance degradation. The current state of magnetic materials depends not only on the immediate applied magnetic field but is also closely related to the entire magnetization history experienced – this is the physical root of hysteresis phenomena. Neural networks’ difficult-to-overcome pain point is the inherent defects of gradient vanishing and long-range dependency modeling. For architectures like Mamba, when facing B(t) and H(t) waveforms of up to 1024 time steps, information from early time steps gradually attenuates after multi-layer propagation, while the essence of hysteresis phenomena requires the model to “remember” key state transitions throughout the magnetization cycle. Therefore, the memory matrix doesn’t simply extend sequence processing capability but constructs a trainable external memory to save typical magnetization behaviors under different operating conditions, allowing current states to directly match similarity with stored historical patterns.

After removing the Top-K feature selection mechanism, overall model performance also degrades significantly. As mentioned earlier, B(t) and H(t) have consistent trends but locally misaligned feature segments, and such transition points often require special attention. Understanding from physical essence, the magnetization process is a highly nonlinear phase transition process. In physical processes, when the applied magnetic field approaches coercivity, numerous domain walls break through pinning positions, leading to sharp changes in magnetization intensity; when approaching saturation magnetization, remaining reverse domains gradually disappear, and the system enters another stable state. That is, key information often concentrates at critical points where system states undergo qualitative changes. Therefore, through learned attention weights, the model spontaneously concentrates attention near inflection points of hysteresis loops, avoiding neglect of the most critical information points.

Removal of the multi-objective optimization framework also leads to model performance degradation, reflecting common misconceptions about core loss prediction tasks. The huge dynamic range of core loss data spanning 6 orders of magnitude causes traditional MSE loss functions to face serious gradient imbalance problems – models tend to prioritize fitting high-loss samples while ignoring low-loss regions that occupy major application scenarios. Since conventional loss functions generally measure specific values like MSE or RMSE, the contribution difference between high-loss and low-loss samples to conventional loss functions is exponential. This also explains why errors exceeding 100% appeared in maximum values during K-fold validation – the optimization process is almost completely dominated by high-loss samples. Our multi-objective framework actually achieves a kind of “dynamic range compression” in feature space. Through the synergistic effects of multiple loss functions, loss values originally spanning 6 orders of magnitude in numerical space are mapped to more balanced gradient distributions in optimization space. This compression isn’t simple logarithmic transformation but an adaptive, physics-preserving nonlinear mapping.

Based on the ablation experimental results in Table 10, we can quantitatively evaluate the contribution of each component. Taking N87 material at 25°C as an example, the complete model achieves an Error RMS of 3.78%. After removing the memory module, Error RMS increases to 4.98%, representing a 31.7% performance degradation, indicating that the memory enhancement mechanism contributes most significantly to overall performance. This validates the critical role of external memory in storing and retrieving typical magnetization patterns. After removing Top-K selection, Error RMS becomes 4.76%, showing a 25.9% performance degradation, demonstrating that the intelligent feature selection mechanism is the second-largest contributing factor. It effectively addresses the temporal mismatch problem between B(t)B(t) B(t) and H(t)H(t) H(t) by focusing on key time points. After removing the multi-objective loss, Error RMS becomes 4.09%, with an 8.2% performance degradation. Although the contribution is relatively smaller, it remains non-negligible, as this loss function guides the model to learn more reasonable internal representations through physical constraints. Using the pure Mamba baseline (removing all innovative components) yields an Error RMS of 5.02%, which is 32.8% worse than the complete model. This indicates that the synergistic effect of the three components is slightly better than simple linear superposition (31.7% + 25.9% + 8.2% = 65.8% > 32.8%), demonstrating the holistic nature of the architectural design and positive interactions among components. Similar trends are observed on 3C90 material, where the memory module contributes 34.0% (reducing from 3.67% to 4.92%), Top-K selection contributes 29.2% (reducing to 4.76%), and multi-objective loss contributes 10.9% (reducing to 4.09%), with the importance ranking of the three components remaining consistent. Across different temperature conditions, the relative contribution ratios of each component remain stable, with the memory module consistently being the most critical component, contributing approximately 30–35% performance improvement on average, Top-K selection contributing 25–30%, and multi-objective loss contributing 8–12%. This consistency further demonstrates the robustness of the EMA-Mamba architecture design and the clarity of each component’s functionality.

#### Comparative experiments

Previously, we analyzed that empirical formulas have flexibility issues under temperature and other conditions compared to neural networks, leading to poor accuracy and adaptability. Therefore, this section comprehensively evaluates the performance advantages of EMA-Mamba in core loss prediction tasks by systematically comparing it with various representative neural network methods. All methods adopted the same training strategy and data division to ensure fair comparison. Table 11 shows the comprehensive performance comparison of various neural network methods on the MagNet dataset.

**Table 11:** Comprehensive performance comparison of different neural network methods.

Method	R² (%)	Error RMS (%)	Error 95-Pct (%)	Error max (%)
Random Forest	98.45	12.78	45.23	187.34
XGBoost	98.89	10.34	38.67	165.89
SVR	97.23	18.45	62.34	223.45
LSTM	99.74	8.92	32.45	134.56
GRU	99.71	9.34	34.67	142.78
TCN	99.68	9.78	36.89	148.23
Transformer	99.82	7.45	28.34	118.67
Informer	98.36	7.32	29.56	141.34
PINN	99.79	8.12	30.78	125.89
Mamba	99.86	6.84	26.22	102.34
EMA-Mamba (Ours)	99.95	4.50	16.41	78.23

From the results, it can be seen that traditional machine learning methods have Error avg ranging from 10% to 18%, with ${\text{R}}^{\text{2}}$ below 99%, indicating that simple non-deep learning regressors struggle to capture the complex nonlinear dependencies and temporal dynamics in 1024-step waveforms. RNN-based models (LSTM and GRU) achieve Error avg of approximately 9%. Although they can process temporal information, they suffer from gradient vanishing and long-range dependency modeling difficulties on 1024-step sequences. TCN expands the receptive field through dilated convolution, but its Error avg remains at 9.78%, suggesting that pure convolutional architectures have limitations in global temporal modeling. Transformer, leveraging the self-attention mechanism, achieves an Error avg of 7.45%, outperforming RNN and CNN series, but its $O({\text{n}}^{\text{2}})$ computational complexity imposes a significant computational burden on 1024-step inputs. PINN achieves an Error avg of 8.12% by incorporating physical constraints, demonstrating that the integration of physical knowledge helps improve prediction accuracy, but its simplified assumptions in physical modeling limit further performance gains. The original Mamba model achieves an Error avg of 6.84% and ${\text{R}}^{\text{2}}$ of 99.86%, proving the inherent advantage of state space models in long sequence modeling, with its linear complexity enabling efficient processing of long waveform inputs. Building on this, EMA-Mamba reduces Error avg to 4.50%, representing a 34.2% improvement over the original Mamba. Error 95-Pct decreases from 26.22% to 16.41%, an improvement of 37.4%. This significant enhancement explicitly stems from the memory enhancement mechanism’s adaptive storage and retrieval capability for typical magnetization patterns, Top-K attention’s intelligent selection of key time points, and the multi-objective loss function’s fine-grained decoupled modeling of different loss mechanisms, rather than simple model architecture switching.

To rigorously verify whether the performance improvement of EMA-Mamba over baseline methods is statistically significant rather than random fluctuation, we conducted paired t-tests based on 5-fold cross-validation results. The paired t-test is a standard statistical method for evaluating the significance of performance differences between two methods on the same data folds. By calculating performance differences on each fold and testing whether the mean of these differences significantly deviates from zero, random factors causing performance fluctuations can be ruled out. Statistical test results in Table 12 show that the improvements of EMA-Mamba over all baseline methods are highly statistically significant (p<0.01), demonstrating that the performance gains are not due to random fluctuations.

**Table 12:** Statistical significance testing (Paired t-test based on 5-fold cross-validation).

Comparison Method	Mean Error avg Difference (%)	t-value	p-value	95% Confidence Interval (%)
EMA-Mamba vs iGSE	6.34	12.78	< 0.001	[5.12, 7.56]
EMA-Mamba vs LSTM	4.42	9.23	< 0.001	[3.45, 5.39]
EMA-Mamb avs Transformer	2.95	7.89	0.001	[2.01, 3.89]
EMA-Mamba vs PINN	3.62	8.34	< 0.001	[2.78, 4.46]
EMA-Mamba vs Mamba	2.34	6.12	0.002	[1.56, 3.12]

In modeling complex physical systems, simply increasing model capacity or using more advanced network structures doesn’t necessarily bring performance improvements. The key lies in whether the network architecture can naturally express the physical essence of the problem. EMA-Mamba achieves precise modeling of the complex physical process of core loss prediction by combining memory augmentation mechanisms, intelligent feature selection mechanisms, and multi-objective optimization frameworks. Under all test conditions, the model achieves the lowest prediction error and highest coefficient of determination, with significantly better robustness under extreme operating conditions than other methods. From static mapping to dynamic memory, from single-objective optimization to multi-scale collaboration, EMA-Mamba opens new research directions for intelligent modeling of magnetic materials.

## Summary

This research addresses the core challenge of core loss prediction in the power electronics field, proposing an innovative modeling paradigm that integrates physical mechanisms with deep learning. Through in-depth analysis of limitations in traditional methods and shortcomings in existing deep learning approaches, we identified three key technical challenges: long-range temporal dependency modeling, heterogeneous waveform alignment, and multi-scale loss mechanism coordination.

The core innovation of the EMA-Mamba model lies in transforming the physical characteristics of magnetic materials into guiding principles for network architecture design. The memory augmentation mechanism is not simple parameter expansion but a mathematical abstraction of the “memory effect” of magnetic materials – achieving persistent storage and rapid retrieval of historical magnetization states through external memory matrices, enabling the model to “remember” typical behavior patterns under different operating conditions like an experienced engineer. The intelligent feature selection mechanism embodies precise grasp of key physical events in the magnetization process, automatically locating saturation regions and turning points of hysteresis loops through attention mechanisms. Although these regions account for a minimal proportion in the time dimension, they determine the main part of loss characteristics. The multi-objective optimization framework achieves a leap from single numerical fitting to multi-physical mechanism collaborative modeling, balancing contributions from different loss mechanisms within a unified framework through clever loss function design.

Experimental validation fully demonstrates the superiority of EMA-Mamba. The model not only significantly leads in overall performance metrics but more importantly shows excellent stability and generalization capability. Temperature robustness analysis reveals that the model has learned the essential laws of temperature effects rather than surface correlations; material similarity analysis confirms that the model can identify and utilize intrinsic physical connections between different materials for knowledge transfer; ablation experiments clearly show the synergistic effects of various innovative components.

To validate the physical significance of the Top-K mechanism, we conducted a statistical analysis of the temporal positions of B-H curve inflection points. We found that these points are mainly distributed in high-curvature regions of the closed-loop curves, including starting points, inflection points associated with hysteresis loops, and turning points. This aligns with the corresponding relationships of key physical events. Regarding the physical interpretation of memory, we performed statistical analysis on the memory after training and found that the 25 memory slots effectively formed 5 clusters corresponding to low-frequency-low-temperature, low-frequency-medium-temperature,low-frequency-high-temperature,medium-frequency-medium-temperature, high-frequency-medium-temperature, and extreme working conditions, respectively. This clustering pattern reflects the aggregation characteristics of the loss mechanism in the working condition space. Furthermore, we conducted ablation experiments (by removing ${\text{P}}_{\text{f}}\propto \text{f}$, ${\text{P}}_{\text{B}}\propto {\text{B}}^{\text{2}}$, and ${\text{P}}_{\text{f}}$ and substituting them with random noise) to verify that the multi-objective loss function indeed guides the model to decouple the physical mechanisms of frequency loss (dominated by f) and hysteresis loss (dominated by larger B values). The results show that EMA-Mamba surpasses 98% on these physical constraint test samples, demonstrating its capability to generalize beyond physical constraints. This validates that the analysis of this decomposition requires more systematic visualization and deeper insights into physical mechanisms and interpretability. Future work will focus on developing more refined interpretability tools, building memory slot visualization tools that correspond to physical states, and introducing interpretable physical constraints (such as those already achieved in this paper) to meet the specific needs of engineering applications.

The significance of this work lies not only in providing a high-precision magnetic core loss prediction tool, but more importantly in establishing a physics-guided deep learning paradigm. This paradigm provides a replicable methodology for addressing similar complex physical mechanism engineering problems, such as: how to innovate neural network architectures by transforming domain knowledge; how to balance data-driven approaches with physical constraints; how to handle multi-scale coupling phenomena. Future research directions include: extending the framework to other types of magnetic materials and more extreme operating conditions; exploring the interpretability of memory models; establishing mapping relationships between network parameters and physical parameters; discussing the scaling law of magnetic core loss and the relationship between data volume and model performance to provide more refined cross-material adaptability and improve the model’s generalization capability in practical engineering. In conclusion, based on the magnetic component optimization design system of EMA-Mamba, we can achieve adaptive selection and structural design intelligence optimization of material selection in practical engineering applications.
